# Using CEST NMR to discover previously unobserved states on the free energy surface of proteins: Application to the L99A cavity mutant of T4 lysozyme

**DOI:** 10.1016/j.jbc.2025.110989

**Published:** 2025-11-27

**Authors:** Ved Prakash Tiwari, Nihar Pradeep Khandave, D. Flemming Hansen, Guillaume Bouvignies, Lewis E. Kay, Pramodh Vallurupalli

**Affiliations:** 1 Tata Institute of Fundamental Research, Hyderabad, Telangana, India; 2 Department of Structural and Molecular Biology, Division of Biosciences, University College London, London, UK; 3 The Francis Crick Institute, London, UK; 4 Chimie Physique Chimie du Vivant (CPCV), Departement de chimie, Ecole normale superieure, PSL University, Sorbonne Universite, CNRS, Paris, France; 5 Department of Molecular Genetics, University of Toronto, Toronto, Ontario, Canada; 6 Department of Biochemistry, University of Toronto, Toronto, Ontario, Canada; 7 Department of Chemistry, University of Toronto, Toronto, Ontario, Canada; 8 Program in Molecular Medicine, Hospital for Sick Children Research Institute, Toronto, Ontario, Canada

**Keywords:** CEST, CPMG, excited conformational states, free energy surface, protein conformational dynamics

## Abstract

Carr-Purcell-Meiboom-Gill (CPMG) relaxation dispersion experiments establish that at room temperature, the L99A cavity mutant of T4 lysozyme (L99A T4L) interconverts between two compact folded conformations on the millisecond timescale. These include the native state in which the sidechain of Phe114 is exposed to solvent (E state) and a near-native minor state in which the aromatic moiety of Phe114 is buried in the core of the protein (B state, ∼2%). Molecular dynamics simulations that have captured the E to B interconversion in L99A T4L and related mutants suggest that these proteins adopt compact folded conformations other than E and B, yet extensive CPMG studies have not detected such states. In an effort to detect these more elusive conformers experimentally, we have recorded Chemical Exchange Saturation Transfer (CEST) experiments, as the widths of minor state dips in the resulting CEST profiles are sensitive to additional, even more sparse, conformers. Analysis of amide ^15^N and ^13^CH_3_ CEST profiles recorded on L99A T4L show that in addition to states E and B, a rare state (I) populated to ∼0.2% (11.5 °C) exchanges rapidly with state B. CEST-based urea m-values establish that all three states are compact, with interconversion between them proceeding *via* compact transition states. This study highlights the utility of CEST to characterize the free energy surface of a protein by detecting states with a wide range of lifetimes (100 ms to 100 μs) in ways that are not possible using other relaxation-based NMR techniques.

At physiological temperatures protein molecules transition between different conformational states, some of which play crucial roles in function, as well as in processes such as folding, misfolding, and aggregation (1, 2). Molecular function is, thus, predicated on the interplay between an array of diverse conformational states and how these interconvert with one another. The populations of such states are determined by their free energies, while the rates at which two states interconvert with each other depend on the height of the free energy barrier that separates them. Characterization of the free energy surfaces (FESs) of proteins is, therefore, an active field of research, involving a range of experimental and computational methods (2, 3, 4, 5, 6, 7, 8). NMR spectroscopy is a particularly powerful technique for the study of biomolecular conformational dynamics as it can be used to detect motions over the microsecond to second time-scale at almost every site in the molecule (2, 9, 10, 11, 12, 13). Often, the dynamics involve a dominant ground state that is populated to more than ∼90%, which exchanges with different minor conformational states. Due to their low populations and short lifetimes, these minor states are often not visible in regular NMR spectra (14). Consequently, experiments of the Carr-Purcell-Meiboom-Gill (CPMG) (15, 16, 17), *R*_*1ρ*_ (18, 19), Chemical Exchange Saturation Transfer (CEST) (20, 21, 22, 23), or Dark State Exchange Saturation Transfer (DEST) (9, 24) variety, that manipulate magnetization of the visible major state to detect invisible minor states, are used to study conformational exchange. A common feature of all these NMR experiments is the presence of a relaxation delay (*T*_*EX*_) during which exchange occurs, with the exchange quantified through the application of radiofrequency pulses, either continuous or separated by delays, that modulate intensities or linewidths of magnetization from the visible state. The resulting datasets are then analyzed to characterize the underlying exchange process(es). Often chemical shift changes accompany the exchange between states, but this is not a prerequisite for some of the experiments, where minor states can be detected in the absence of such shift differences so long as the magnetization from the sites probed have significantly different transverse relaxation rates ($R_{2}$) in the minor and major conformations (9, 25, 26). Minor states populated typically to as low as ∼0.5% are detected in a variety of exchange processes in proteins (6, 27, 28, 29, 30, 31, 32), nucleic acids (19, 33), and small molecules (34).

Over the last 3 decades the L99A cavity mutant of T4 lysozyme (L99A T4L) has emerged as a model system to study protein conformational dynamics (5, 35, 36, 37, 38, 39, 40). The L99A mutation gives rise to an ∼150 Å^3^ cavity buried in the C terminal domain of the protein, but it does not affect the structure of the native state of the molecule (41). In the lowest energy conformation (native state) of the protein, this cavity is buried with no direct path from the solvent reaching it. Nevertheless, hydrophobic molecules such as benzene can bind the cavity rapidly (37, 41) implying that the protein is dynamic, transiently adopting (higher energy) conformations in which there are pathways for the ligands from solvent to bind. The exact mechanism(s) by which this occurs continues to be investigated using various computational techniques (5, 42, 43, 44). CPMG-based relaxation dispersion studies carried out 2 decades ago to understand ligand binding in this system established the existence of an exchange process in the vicinity of the cavity, which turned out to be unrelated to the binding event. This process, which will be investigated further here, involves an interconversion between the native state and a minor conformer, the latter with a fractional population of ∼2.5% and a lifetime of ∼1.25 ms at 20 °C (36). The structure of the minor state was subsequently determined using CPMG-based data, showing that it is compact and folded, with a similar conformation to the native protein (38) (Fig. S1). The main differences in the structures are localized to the region around Phe114, where the backbone torsion angle ψ of this residue is changed from ∼ +55° in the native state to a helical value of ∼ −50° in the minor state. This change in dihedral angle results in the merging of helices f and g to form a long helix with a concomitant change in the orientation of the sidechain of Phe114 from a solvent exposed pose in the native state to one where the aromatic sidechain is inserted into the cavity in the minor state (Fig. S1). In what follows, therefore, the native state of L99A T4L is referred to as the exposed (E) conformer, as the sidechain of Phe114 is exposed to solvent, and the minor state is denoted as the buried (B) conformer, as the sidechain of Phe114 is within the cavity created by the L99A mutation. Notably, the B state cannot bind hydrophobic ligands as the cavity is occupied by the sidechain of Phe114. Since CPMG experiments could be interpreted to within experimental error on the basis of a simple, two-state E↔B interconversion, molecular dynamics studies were performed to obtain further insight into the E to B exchange process (5, 45, 46, 47). These computational studies suggested that the free energy surface of the protein is rugged, with the protein populating near native folded states other than B and E and that the E↔B interconversion occurs without any unfolding *via* both direct and indirect (*i.e.*, involving intermediates) pathways. There is, however, no direct experimental evidence supporting this level of ruggedness, as states other than B and E have not been observed at 25 °C or lower temperatures despite detailed investigations using CPMG-based NMR experiments over the last several years (16, 36, 38, 48, 49, 50, 51, 52, 53, 54).

CEST experiments can be used to study protein conformational exchange processes occurring on the ∼0.1 to ∼100 millisecond timescale (12, 55, 56, 57, 58), and we showed recently that the widths of minor state dips in CEST profiles, arising from the slow interconversion between major and sparse conformations, can be used to inform on even more sparsely populated states that are in intermediate to fast exchange with the minor conformer (6, 8, 56, 59). That is, the minor state, that itself cannot be directly observed in regular NMR spectra and is detected *via* CEST, can be used to ‘spy’ on even more sparsely populated states. Here we have used this approach to investigate the E to B exchange process in L99A T4L using amide ^15^N and methyl ^13^C CEST experiments (11.5 °C). These experiments establish that L99A T4L populates a second minor state (I, ∼0.2% fractional population) that is in rapid exchange with B, providing experimental evidence of a more complex FES than previously established by CPMG studies, and consistent with MD simulations. Urea m-values of E, I, and B and the transition states connecting them, obtained through analysis of ^15^N CEST data recorded on samples with varying concentrations of denaturant, indicate that state I is as compact as states E and B, and that interconversion between these conformers proceeds *via* compact transition states.

## Results and discussion

### CEST *vs* CPMG for exploring rugged protein energy landscapes

**Figure 1:**
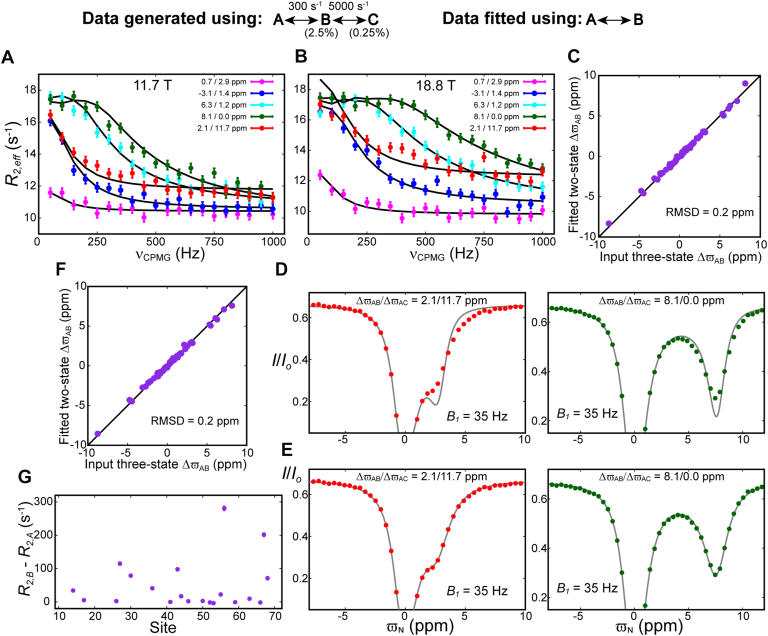
**Minor states of interest that may go undetected in CPMG experiments can often be detected *via* CEST.***A* and *B*, calculated ^15^N CPMG relaxation dispersion curves, $B_{0}$ = 11.7 T (*A*) and 18.8 T (*B*) and *T*_*EX*_ = 20 ms, for a three-state exchange process ($k_{ex,AB}$ = 300 s^−1^, $k_{ex,BC}$ = 5000 s^−1^ and $p_{B}$ = 2.5%, $p_{C}$ = 0.25%) using different sets of ${\Delta \varpi }_{AB}$ and ${\Delta \varpi }_{AC}$ values. In *panels* (*A*) and (*B*) the synthetic CPMG data points are represented using colored circles, while the two-state best-fit to the data ($k_{ex,AB}$ = 292 ± 7 s^−1^ and $p_{B}$ = 2.5 ± 0.1%, $\chi_{red}^{2}$ = 1) is shown using *black lines*. Chemical shift differences, ${\Delta \varpi }_{AB}$ and ${\Delta \varpi }_{AC}$, used to generate the synthetic CPMG profiles shown in (*A*) and (*B*) are listed in the *panels* as ${\Delta \varpi }_{AB}$/ ${\Delta \varpi }_{AC}$. Values of (${\Delta \varpi }_{AB}$, ${\Delta \varpi }_{AC}$) were set to (${\Delta \varpi }_{FI1}$, ${\Delta \varpi }_{FI2}$), obtained from fits of CEST profiles measured at 58 sites of the A39G FF domain (6) that folds from the unfolded to the native state (F) *via* intermediates I1 and I2. *C*, comparison of ${\Delta \varpi }_{AB}$ values used to generate the three-state exchange CPMG data with the corresponding ${\Delta \varpi }_{AB}$ shift differences from two-state fits of the resulting CPMG profiles. The signs of the CPMG derived (two-state) ${\Delta \varpi }_{AB}$ values were assumed to be the same as those used to generate the data. *D and E*, selected synthetic ^15^N CEST profiles along with two-state fits; profiles were computed for $B_{1}$*/T*_*EX*_ = 17Hz/450 ms and 35Hz/400 ms, $B_{0}$ = 18.8 T, and generated using the same three-state exchange parameters used for the CPMG data. Fits carried out with (*D*) $R_{2,B}$ = $R_{2,A}$ ($\chi_{red}^{2}$ = 2.2) and without (*E*) any constraint on $R_{2,B}$ ($k_{ex,AB}$ = 277 ± 2 s^−1^ and $p_{B}$ = 2.8 ± 0.01%, $\chi_{red}^{2}$ = 1). The simulated data are shown using circles, while the grey lines are calculated using the best-fit parameters. The uncertainties in the $I/I_{0}$ values are smaller than the size of the filled circles. *F*, comparison between the ${\Delta \varpi }_{AB}$ values used to generate the CEST data for the three-state exchange process and those obtained from fits of a two-state exchange model to the data. *G*, very large best-fit $R_{2,B}$ values are obtained when a two-state model with $R_{2,B}$ unconstrained is fit to the synthetic three-state ^15^N CEST data; ($R_{2,B}$ - $R_{2,A}$) values are only shown for sites for which $\left|{\Delta \varpi }_{AB}\right|$ > 2.0 ppm.

Both CEST and CPMG relaxation experiments are used to probe FESs of biomolecules, providing insights into functional dynamics that are not available from other biophysical techniques (2, 6, 8, 32, 60, 61). CPMG-based dispersion profiles of the L99A T4L cavity mutant were well fit to a two-state equilibrium involving the interconversion of folded states E and B (16, 36, 38, 48, 49, 50, 51, 52, 53, 54), as described in the Introduction, but further insight into the energy landscape of L99A T4L could not be obtained as additional states were not detected. Yet MD simulations showed a rugged FES, with multiple folded conformations. In an effort to obtain experimental support for states beyond E and B, we wondered whether CEST experiments might prove to be more useful than those based on CPMG relaxation dispersion. To address this possibility, initially computationally, we considered a linear three-state exchange process A↔B↔C involving a major state A, and two minor states, B and C, with $p_{A}$ (97.25%) >> $p_{B}$ (2.5%) >> $p_{C}$ (0.25%) and exchange rates $k_{ex,AB}$ = 300 s^−1^ and $k_{ex,BC}$ = 5000 s^−1^. Here $p_{i}$ is the fractional population of state *i* and $k_{ex,ij}$ = $k_{ij}$ + $k_{ji}$ is the sum of the forward and reverse rates for the i↔j interconversion process. Synthetic CPMG data were generated for 58 combinations of $\Delta \varpi_{AB}$ and $\Delta \varpi_{AC}$ values (see legend to Fig. 1), where $\Delta \varpi_{ij}$ = $\varpi_{j}$ – $\varpi_{i}$ and $\varpi_{i}$ and $\varpi_{j}$ are the chemical shifts (ppm) of the spin in question in states *i* and *j*, respectively. The shift differences considered in this analysis are derived from experimental values obtained in a study of the folding of the A39G FF protein domain (6), and it has been assumed that the longitudinal (*R*_*1*_) and transverse (*R*_*2*_) relaxation rates of a given spin in each of the exchanging states are identical, $R_{1,A}$ = $R_{1,B}$ = $R_{1,C}$ = 1 s^−1^ and $R_{2,A}$ = $R_{2,B}$ = $R_{2,C}$ = 10 s^−1^. Five representative calculated ^15^N CPMG relaxation dispersion profiles are shown in Figure 1*A* (11.7T/500 MHz) and 1*B* (18.8T/800 MHz) in which the effective transverse relaxation rates, $R_{2,eff}$, are denoted by filled circles. Interestingly, the synthetic CPMG datasets generated using the three-state exchange model can be fit ($\chi_{red}^{2}$ ∼1.1) using a simple two state A↔B exchange process with $k_{ex,AB}$ = 292 ± 7 s^−1^ and $p_{B}$ = 2.5 ± 0.1%. The two-state (A↔B) fits to the three state CPMG data were carried out by assuming $R_{2,A}$ = $R_{2,B}$, and are shown with black lines in Figure 1, *A* and *B*. Visual inspection of all the fits shows that they are reasonable, and since $\chi_{red}^{2}$ ∼1.1 there is no need to consider a more complicated exchange model to account for the data. Notably, the $\Delta \varpi_{AB}$ values obtained from the two-state fit are in very good agreement (RMSD 0.2 ppm, Fig. 1*C*) with the $\Delta \varpi_{AB}$ values used to generate the three-state data, further establishing that the CPMG experiment is very insensitive to the presence of state C, most likely due to its low population and short lifetime. It is worth noting that in the example above, and in those following below, we fit two-state models of exchange (A↔B) to three-state exchange data (for example A↔B↔C). We do not mean to imply that A and B conformers in the two-state exchange model correspond to A and B, respectively, in the three-state model, although they often do, depending on exchange rates and populations. The extracted exchange parameters ($k_{ex,AB}$ = 292 s^−1^ and $p_{B}$ = 2.5%) from the two-state fits of the simulated data are similar to those obtained from analysis of experimental data reporting on the L99A T4L E ↔ B interconversion process at ∼15 °C ($k_{ex,EB}$ ∼400 s^−1^ and $p_{B}$ ∼2%) (36). Hence, states A and B in Figure 1 can be ‘likened’ to the E and B states of L99A T4L, respectively, while C corresponds to an additional minor state that cannot be detected by CPMG methods. The simulations of Figure 1, *A*–*C*, thus, rationalize why it has not been possible to observe conformers beyond E and B in our previous CPMG-based studies of L99A T4L.

Although CPMG experiments are typically preferred over their CEST counterparts for studies of μs – low ms exchange processes, it is now clear that CEST can be used to study dynamics over a wider timescale window (100 ms to 100 μs) than originally thought (100 ms to 5 ms). Indeed, in contrast to simulations of CPMG data discussed above where state C could not be detected, the situation is more favorable when one considers similar simulations of CEST profiles (6, 59). Synthetic CEST datasets (weak *B*_*1*_ fields of 17 and 35 Hz; static magnetic field of 18.8T) were computed for an A↔B↔C exchange scheme, using the same exchange parameters and combinations of $\Delta \varpi_{AB}$ and $\Delta \varpi_{AC}$ values that were used to generate the CPMG data above. The resulting CEST profiles were then fit to a two-state A↔B exchange model and the data were only poorly reproduced when the condition $R_{2,A}$ = $R_{2,B}$ was enforced ($\chi_{red}^{2}$ = 2.2), Figure 1*D*. However, the quality of the fits was much better ($\chi_{red}^{2}$ = 1) when an A↔B exchange model was used in which both $R_{2,A}$ and $R_{2,B}$ were independent fitting parameters, with best fit values of $k_{ex,AB}$ = 277 ± 2 s^−1^ and $p_{B}$ = 2.8 ± 0.01% (Fig. 1*E*). The fitted two-state $\Delta \varpi_{AB}$ values are once again in good agreement (RMSD 0.2 ppm) with those used to generate the data (Fig. 1*F*), but $R_{2,B}$ values are significantly elevated, with some larger than 75 s^−1^ compared to $R_{2,B}$ = 10 s^−1^ used to generate the profiles (Fig. 1*G*). It is noteworthy that, as in the analysis of the synthetic CPMG data, fitted chemical shift values from CEST datasets that appear consistent with expectations (in the present case ‘correct’ $\Delta \varpi_{AB}$ values were obtained from fits to the ‘incorrect’ two-state model) cannot be used as an argument for the legitimacy of an assumed kinetic scheme. Rather, the elevated extracted transverse relaxation rates in Figure 1*G* are a telltale sign of a more complex exchange mechanism than that assumed, whereby the dips derived from state B are broadened due to exchange with an additional sparse state C (6, 20, 59, 62). In the analysis of experimental data, it is, in general, relatively straightforward to establish that the extracted relaxation rates (which in principle should be intrinsic relaxation values) are larger than expected, and hence reporting on a more complex exchange scheme than that used in fits, since $R_{2}$ values can be estimated from the size of the complex studied (6, 20, 59, 62). Thus, CEST profiles can provide information on multi-state exchange processes even when only a single minor exchange dip is observed and when such information is not available from the analysis of CPMG data. However, one must exercise caution while applying the above strategy to coupled spins, as in the case of ^13^C CEST data recorded on uniformly ^13^C labeled samples or ^1^H CEST data because the shapes of the resulting CEST profiles are affected by $J_{CC}$ couplings or ^1^H−^1^H dipolar interactions respectively.

### Application of CEST to study of the FES of L99A T4L

**Figure 2:**
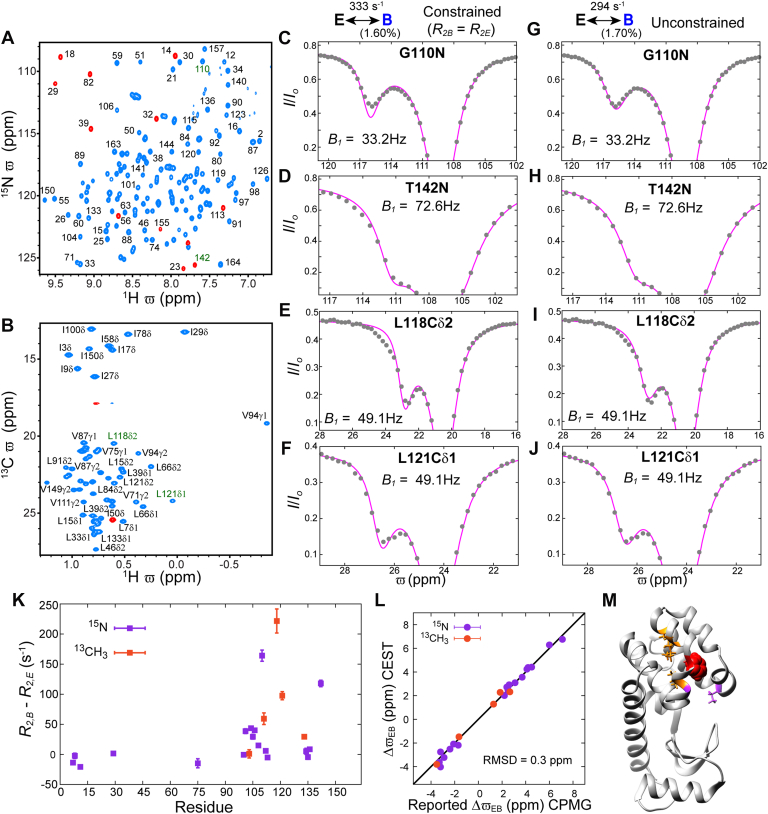
**The ‘invisible’ B state of L99A T4L interconverts with even more sparsely populated state(s).** Amide ^1^H-^15^N (*A*) and ILV methyl ^1^H-^13^C (*B*) HSQC spectra of L99A T4L (11.5 °C; 16.4 T). Only peaks from the major (E) state are visible in the spectra and (some well resolved peaks) are labelled according to the amide or methyl site from which they arise. Aliased peaks are shown in *red*. (*C*–*J*) Representative amide ^15^N CEST profiles from G110 (*C*, *G*) and T142 (*D*, *H*), along with methyl ^13^C CEST profiles from L118Cδ2 (*E*, *I*) and L121Cδ1 (*F*, *J*). Correlations arising from G110/T142 and L118Cδ2/L121Cδ1 are indicated in *green* in (*A*) and (*B*), respectively. A two-state (E↔B) model does not account for the CEST data when $R_{2,B}$ = $R_{2,E}$ (*C*–*F*), however, noticeably better fits are obtained when $R_{2,B}$ is not constrained (*G*–*J*). The experimental data points are shown using *grey circles*, while the *magenta lines* are calculated using the best-fit parameters. The uncertainties in the $I/I_{0}$ values are smaller than the size of the filled circles. *K*, amide ^15^N and methyl ^13^C $R_{2,B}$ - $R_{2,E}$ values obtained from two-state fits where $R_{2,B}$ is not constrained; only values for amide ^15^N sites with $\left|{\Delta \varpi }_{EB}\right|$ > 2.0 ppm and for methyl ^13^C sites with $\left|{\Delta \varpi }_{EB}\right|$ > 1.0 ppm are shown. *L*, comparison of ${\Delta \varpi }_{EB}$ values obtained from the two-state analysis of the amide ^15^N and methyl ^13^CH_3_ CEST data with corresponding shift differences obtained previously *via* analysis of CPMG-based datasets (38, 54). Signs for the CPMG-derived methyl ^13^C ${\Delta \varpi }_{EB}$ values are not available and were assumed to be the same as those derived from the CEST experiments. *M*, ribbon representation of L99A T4L [PDB ID: 3DMV, (83)] showing residues with large (>50 s^−1^) amide ^15^N (*purple*) or methyl ^13^CH_3_ (*orange*) $R_{2,B}$ - $R_{2,E}$ values (stick representation). Phe114 is shown in *red* (CPK representation).

Having established that it may be possible to detect additional states from analysis of CEST data that have eluded observation when using CPMG experiments we next recorded amide ^15^N and methyl ^13^C CEST datasets using a [U-^15^N; ^2^H; Ileδ1-^13^CH_3_; Leu, Val-^13^CH_3_/^12^CD_3_] labeled L99A T4L sample at 11.5 °C (Fig. 2, *A*–*J*; 16.4 T). Several CEST profiles showed a dip corresponding to a minor state (Fig. 2, *C*–*J*) in addition to one derived from the major conformer, but the ^15^N ($B_{1}$ = 18.1, 33.2, 51.8, 72.6, and 129.6 Hz; $B_{0}$ = 16.4 T) and ^13^CH_3_ ($B_{1}$ = 16.2, 32.4, 49.1, and 98.2 Hz, $B_{0}$ = 16.4 T) data could not be jointly fit to a two-state exchange model in which the major and minor state $R_{2}$ values were constrained to be the same ($\chi_{red}^{2}$ = 2.2, Fig. 2, *C*–*F*). However, a two-state exchange model with no constraint on major and minor state $R_{2}$ values fit the CEST data better ($\chi_{red}^{2}$ = 1.2, Fig. 2, *G*–*J*), albeit with large $R_{2,B}$ values at some sites (Fig. 2*K*). Extracted Δϖ values from these fits are in good agreement with those derived from analysis of CPMG dispersion profiles (RMSD 0.3 ppm, Fig. 2*L*), confirming that the CEST experiments probe the E↔B exchange process as well, with best-fit exchange parameters, $k_{ex,EB}$ = 294 ± 4 s^−1^ and $p_{B}$ = 1.70 ± 0.01%. Importantly the elevated $R_{2,B}$ values (Fig. 2*K*) clearly establish the presence of at least one hitherto undiscovered minor state in exchange with state B. These $R_{2,B}$ values localize around the cavity (Fig. 2*M*), suggesting that residues in this region can adopt conformations other than those associated with the E and B states.

**Figure 3:**
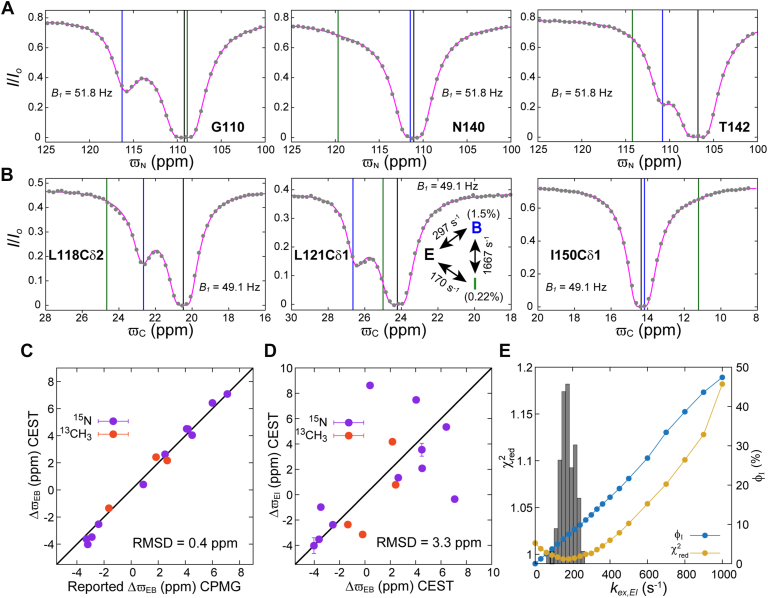
**A three-state exchange model accounts for the L99A T4L CEST data (11.5 °C; 16.4 T).** Fits of the amide ^15^N CEST profiles (*A*) from G110, N140 and T142 and methyl ^13^C (*B*) CEST profiles from L118Cδ2, L121Cδ1, and I150Cδ1 to a triangular model of chemical exchange (inset in the middle panel in (*B*)). *Grey circles* represent experimental data, while the *magenta line* is calculated from the best-fit (three-state) parameters. The uncertainties in the $I/I_{0}$ values are smaller than the size of the filled circles. *Black*, *blue*, and *green vertical lines* are drawn at the best-fit values of $\varpi_{E}$, $\varpi_{B}$, and $\varpi_{I}$, respectively. *C*, comparison of ${\Delta \varpi }_{EB}$ values obtained from the three-state analysis of amide ^15^N and methyl ^13^C CEST data with the corresponding shift differences from analysis of CPMG profiles (38, 54). Signs of the CPMG derived ${\Delta \varpi }_{EB}$ values for the methyl ^13^C sites are not available and are assumed to be the same as those obtained from the CEST experiments. *D*, the poor correlation between ${\Delta \varpi }_{EI}$ and ${\Delta \varpi }_{EB}$ values obtained from the joint three-state analysis of the amide ^15^N and methyl ^13^C CEST profiles establish that states I and B are unique. *E*) $\chi_{\text{red}}^{2}$*vs*$k_{ex,EI}$ (*yellow*), along with the distribution of fitted $k_{ex,EI}$ values from the three-state triangular model (*grey*), based on a joint analysis of the ^15^N and ^13^C CEST data. For each value of $k_{ex,EI}$ the fraction of molecules that convert from E to B *via* I ($\phi_{I}$ = $\left(k_{EI}\frac{k_{IB}}{k_{IE}+k_{IB}}\right)/\left(k_{EB}+k_{EI}\frac{k_{IB}}{k_{IE}+k_{IB}}\right)$) is also shown (*dark blue*). The distribution of fitted $k_{ex,EI}$ values was obtained *via* a bootstrap procedure (81, 82) with 100 trials.

Next, we proceeded to test whether the addition of a single intermediate state, I, to the exchange scheme could account for the increased $R_{2,B}$ rates obtained *via* two-state analyses. The ^15^N ($B_{1}$ = 18.1, 33.2, 51.8, 72.6 & 129.6 Hz; $B_{0}$ = 16.4 T) and methyl ^13^C ($B_{1}$ = 16.2, 32.4, 49.1 & 98.2 Hz, $B_{0}$ = 16.4 T) CEST data were fit to a global triangular three-state exchange model in which E, B, and I exchange with each other subject to the constraint $R_{2,E}$ = $R_{2,B}$ = $R_{2,I}$. In the absence of any information regarding the minor-state chemical shifts, determining the right three-state exchange model from NMR relaxation dispersion data is challenging, often requiring extensive computation (60). However, recently we noted that the correct three-state model can be readily obtained so long as the signs of the chemical shift differences between the minor state resonances (*i.e.* signs of ${\Delta \varpi }_{BI}$ values here) are known (59). This information is captured in a two-state analysis of (low $B_{1}$) CEST data recorded at multiple temperatures (See Supporting Information, Fig. S2), and once available the correct three-state exchange model is obtained without extensive computation by constraining the signs of the chemical shift differences between the minor state resonances (here ${\Delta \varpi }_{BI}\geq 0$ for positive ${\Delta \varpi }_{BI}$ or ${\Delta \varpi }_{BI}\leq 0$ for negative ${\Delta \varpi }_{BI}$) during data fitting (59). In the case of L99A T4L, the three-state model obtained using this procedure fit the amide ^15^N and methyl ^13^C CEST data well, and slightly better than any of the two-state models attempted previously (compare Figure 3, *A* and *B* with Fig. 2, *C*–*J*). Exchange parameters $k_{ex,EB}$ = 297 ± 6 s^−1^, $k_{ex,EI}$ = 170 ± 37 s^−1^, $k_{ex,BI}$ = 1667 ± 44 s^−1^, $p_{B}$ = 1.5 ± 0.01%, and $p_{I}$ = 0.22 ± 0.01% ($\chi_{red}^{2}$ = 1) were obtained, establishing that the conformational dynamics of L99A T4L can be well-modeled as a three-state exchange process involving states E, B, and a single new minor state (I). As expected, based on the simulations of Figure 1, the $\Delta \varpi_{EB}$ values derived from the CEST fits involving the three-state model are in good agreement with the corresponding shift differences obtained previously from two-state fits of CPMG data (16, 38) (RMSD 0.4 ppm, Fig. 3*C*). Moreover, the I state chemicals shifts (Table S4) differ from those of the B state (Fig. 3*D*) accounting for the broadening of the minor-state (B) dips in the CEST profiles. In addition to the triangular model, we also checked whether any of the simpler linear three-state models could explain the CEST data, requiring that $R_{2,E}$ = $R_{2,B}$ = $R_{2,I}$ in all cases. The bifurcated I↔E↔B model, where I and B exchange independently with the major state E, did not fit the data well ($\chi_{red}^{2}$ ∼1.9) as it cannot account for the broadening of the B state dips in the CEST profiles. The linear E↔B↔I model where I is off-pathway does fit the data well ($\chi_{red}^{2}$ = 1.01) (Fig. S3), suggesting that the role of state I in the E↔B interconversion processes is likely small. This is consistent with the shallow minimum that is obtained in the $\chi_{red}^{2}$ *vs* $k_{ex,EI}$ surface for the triangular model (Fig. 3*E*), with a minimum $k_{ex,EI}$ value of ∼200 s^−1^, but where $k_{ex,EI}$ can be quadrupled or quartered with little change in the goodness of fit. Figure 3*E* also plots the fraction of state B that is formed from state E along the E↔I↔B pathway of the triangular model, $\phi_{I}$ (see legend to Fig. 3). Over the range of best-fit $k_{ex,EI}$ values ($\chi_{red}^{2}$ ≤ 1.05) $\phi_{I}$ < 20%, consistent with a relatively small contribution from the I state in the conversion from E to B. As the CEST data indicate (Fig. 3*E*) that the direct (E to B) pathway dominates the interconversion between E and B, it is not surprising that the E↔I↔B linear on-pathway scheme, where the interconversion between E and B proceeds solely *via* I, does not account for the data ($\chi_{red}^{2}$ = 1.3) as well as the triangular and the linear E↔B↔I models ($\chi_{red}^{2}$ ∼1 in both cases). As mentioned in the introduction, MD simulations suggest that the E↔B interconversion proceeds both directly and indirectly *via* intermediates (5, 46). Thus, we prefer the triangular scheme over the E↔B↔I scheme as it supports the notion of both direct and indirect interconversion pathways from E to B (5, 46, 47), as observed in MD simulations (5, 46), with the direct E↔B transition being dominant. It should be noted that both the triangular and the linear E↔B↔I scheme result in very similar chi-squared statistics, minor state populations and chemical shifts (Fig. S3).

**Figure 4:**
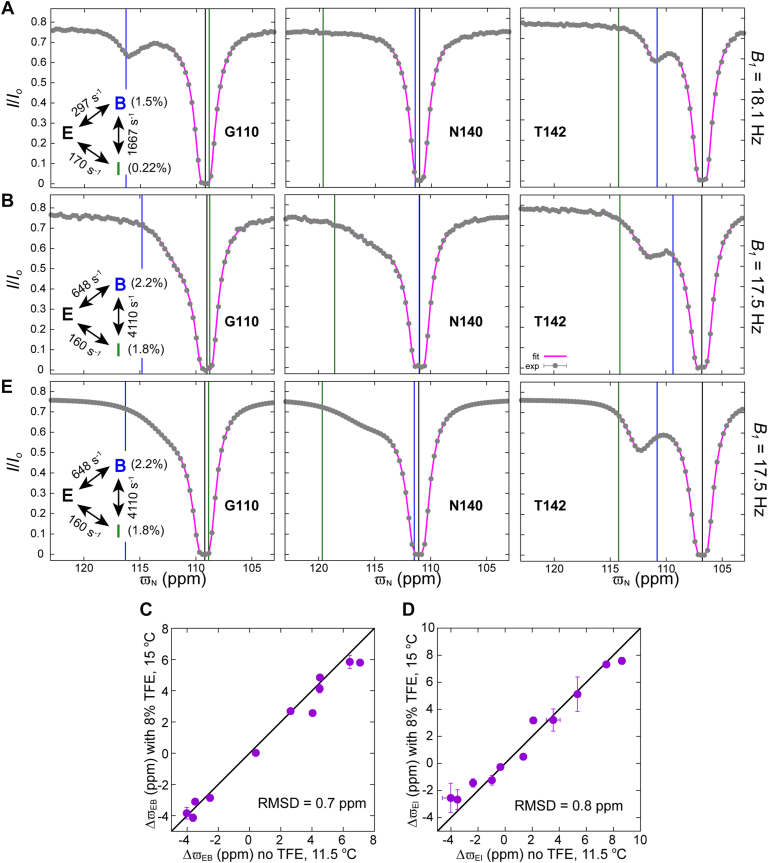
$\varpi_{I}$**values obtained from the three-state analysis of the CEST data are robust.** Amide ^15^N CEST profiles from G110, N140 and T142 measured using samples without (*A*) or in the presence of TFE (8% V/V) (*B*). The CEST data shown in (*A*) and (*B*) are recorded at 11.5 °C and 15 °C, respectively. In (*A*) and (*B*), the best-fit three-state models are shown, along with *black, blue*, and *green vertical lines* drawn at the positions of the best-fit $\varpi_{E}$, $\varpi_{B}$, and $\varpi_{I}$ values, respectively, obtained from the analysis of CEST data recorded in the absence (*A*) and presence (*B*) of TFE. The experimental data are represented using *grey circles*, with the *magenta lines* calculated using the (three-state) best-fit parameters. The uncertainties in the $I/I_{0}$ values are smaller than the size of the filled circles. Comparison of amide ^15^N ${\Delta \varpi }_{EB}$ (*C*) and ${\Delta \varpi }_{EI}$ (*D*) values obtained from the analysis of the CEST data recorded with and without TFE. *E*, ^15^N CEST profiles calculated using the chemical shifts from (*A*) and the exchange parameters indicated in the inset of the *left panel* in (*B*), obtained from fits of the ‘TFE data’, are qualitatively similar to the experimental CEST profiles in (*B*). In (*E*) *black, blue*, and *green vertical lines* are drawn at the positions of the best-fit $\varpi_{E}$, $\varpi_{B}$, and $\varpi_{I}$ values, respectively, obtained in the absence of TFE (*A*).

TFE (2,2,2-Trifluoroethanol) has previously been shown to stabilize certain sparsely populated states including some folding intermediates (63) and it has been used to increase the population of minor states in CEST NMR studies (6). Hence to test if the best-fit $\varpi_{I}$ values are reasonable we added a small fraction of TFE to the sample with the hope of changing the relative populations of the B and I states so as to modify the shapes of the resulting CEST profiles in predictable ways that are consistent with the fitted chemical shifts. Addition of 8% TFE increased $k_{ex,BI}$ to ∼4000 s^−1^ and, more importantly, increased $p_{B}$ and $p_{I}$ to ∼2.2% and ∼1.8%, respectively, relative to values of 1.5 ± 0.01% ($p_{B}$) and 0.22 ± 0.01% ($p_{I}$) in the absence of the additive. ^15^N CEST profiles in the absence and presence of TFE are compared in Figure 4, *A* and *B*, respectively. For G110, where $\varpi_{I}$ ∼ $\varpi_{E}$, the distinct dip at ∼ $\varpi_{B}$ in the absence of TFE broadens significantly when TFE is added and shifts in-between $\varpi_{I}$ and $\varpi_{B}$, as expected. In the case of N140, where $\varpi_{B}$ ∼ $\varpi_{E}$ so that a minor state dip is not visible in the *B*_*1*_ = 18.1 Hz CEST profile in the absence of TFE, a broad dip emerges between $\varpi_{I}$ and $\varpi_{B}$ in the presence of TFE. In the case of T142 where $\varpi_{B}$, $\varpi_{E}$, and $\varpi_{I}$ are all distinct, the minor state dip at ∼ $\varpi_{B}$ in the absence of TFE broadens and shifts to a position between $\varpi_{B}$ and $\varpi_{I}$ when TFE is added. The shifts, broadening, and appearance of dips in these examples are consistent with expectations based on the assignments of chemical shifts in the absence of TFE. Further validation of the obtained chemical shifts is obtained by noting that the fitted $\varpi_{B}$ and $\varpi_{I}$ values generated from samples with and without TFE are similar (Fig. 4, *C* and *D*). Finally, ^15^N CEST profiles calculated using $\varpi_{E}$, $\varpi_{B}$, and $\varpi_{I}$ values from fits of datasets recorded on samples without TFE, but with exchange rates and populations obtained from fits of the TFE-based data (Fig. 4*E*), are qualitatively similar to the ^15^N CEST profiles recorded on samples with TFE (Fig. 4*B*). Taken together, these results establish that the obtained $\varpi_{I}$ values are robust.

With a three-state model of exchange for L99A T4L (11.5 °C) available from fits of CEST data (Fig. 3), it is now possible to understand why additional states beyond E and B could not be observed in CPMG experiments: the low population of state I and its rapid interconversion with state B effectively ‘subsumes’ state I into state B. We have simulated amide ^15^N and methyl ^13^C CPMG dispersion profiles (*T*_*EX*_ = 20 ms, with $\nu_{CPMG}$ varying from 50 to 1000 Hz) at fields of 11.7 T and 18.8 T using CEST parameters (triangular model) obtained from fits of 11 ^15^N and 4 ^13^C sites (Fig. S4). The synthetic CPMG profiles obtained were well fit with a two-state model of conformational exchange that assumed equivalent intrinsic transverse relaxation rates for corresponding spins in each state ($\chi_{red}^{2}$ = 1.04; $k_{ex,EB}$ = 318 ± 45 s^−1^ and $p_{B}$ = 1.5 ± 0.3%).

**Figure 5:**
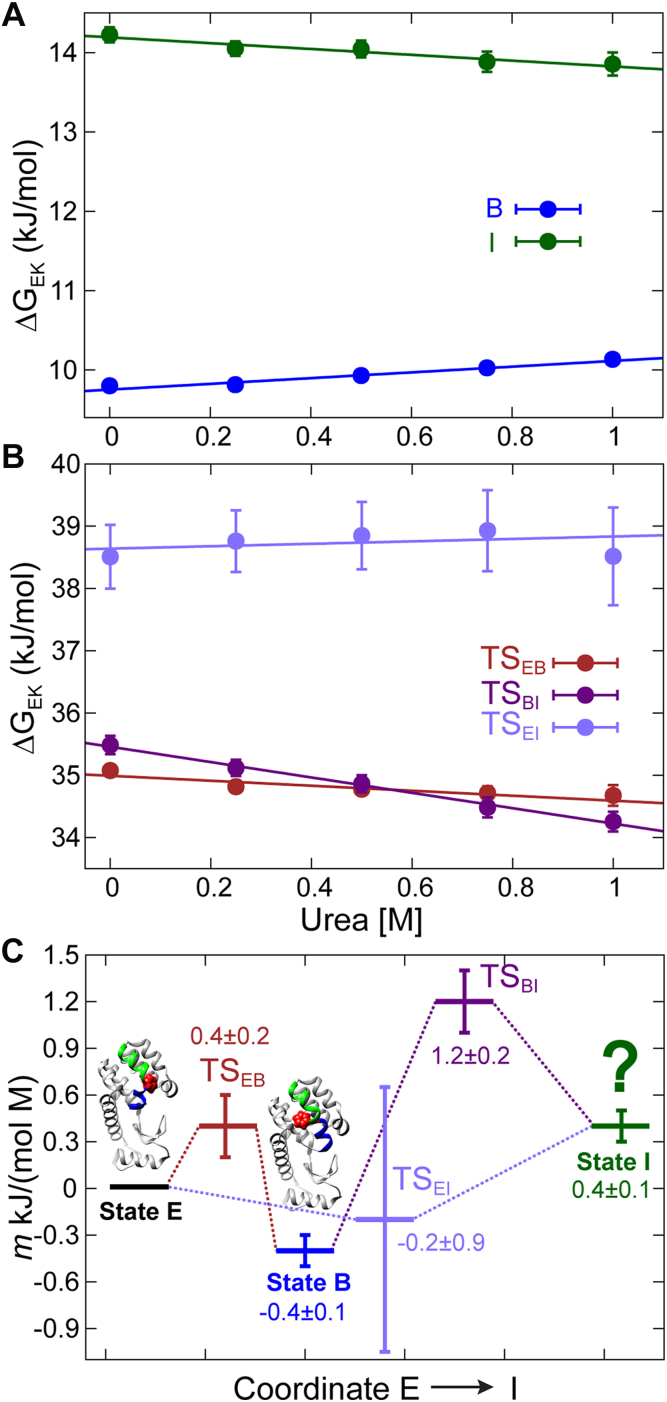
**The FES of L99A T4L in the vicinity of the cavity consists of compact conformations.***A*, the change in free energy of states B and I (with respect to E) as function of urea concentration. For a state K, $\Delta G_{EK}$ is calculated from the populations according to the relation $\Delta G_{EK}=-RTln\left(p_{K}/p_{E}\right)$. *B*, changes in the free-energies of the transition states TS_EB_, TS_BI_, and TS_EI_ as a function of urea concentration. For TS_LM_, $\Delta G_{ETSLM}$ is calculated from rates and populations using the relation $\Delta G_{ETSLM}=-RTln\left(p_{L}/p_{E}\right)-RTln\left(k_{LM}/C\right)$ where C is set to be 10^7^ s^−1^ and does not affect the calculated $m_{TSLM}$ value. *C*, schematic representation of the urea m-value landscape of L99A T4L, 11.5 °C. Structures of L99A T4L in the E [PDB: 3DMV (83)] and B [PDB: 2LCB (38)] states are also shown.

We wondered whether the level of compactness of state, I is similar to states E and B, or whether I is more expanded, as might be expected in the case of a partially folded folding-intermediate (8, 64). It is not possible to address this question from amide ^15^N and methyl ^13^C chemical shifts alone. However, urea m-values (1, 65), that provide a measure of how the free energy (G) of a state changes with respect to a reference state, can be interpreted in terms of the compactness of the queried state, since expanded conformers, such as the unfolded ensemble of a protein, have a large number of urea binding sites leading to a high m-value. Here we define $m_{K}=-\frac{d\Delta G_{EK}}{d\left[urea\right]}$, where E is the reference ground state of L99A T4L, and K denotes either states I or B, or a transition state, TS_LN_, that connects states L and N in which case $m_{TSLN}$ is the m-value of TS_LN_. Urea m-values for states B, I and all possible transition states were obtained by analyzing ^15^N CEST data recorded using L99A T4L samples prepared with varying amounts of urea, 11.5 °C (Figs. 5, S5, Table S5). As expected from the structures of the E and B states, that are both well-folded (38, 41), $m_{B}$ (=−0.4 ± 0.1 kJ mol^−1^ M^−1^) ∼ 0. Similarly, $m_{I}$ (=0.4 ± 0.1 kJ mol^−1^ M^−1^) ∼ 0, so that the level of compactness of state I is similar to states E and B. The m-values of the transition-states are also small. For example, $m_{TSEB}$ (=0.4 ± 0.2 kJ mol^−1^ M^−1^) ∼ 0, consistent with interconversion between E and B without expansion of the protein, in agreement with MD simulations (5, 46). Interconversion between states B and I proceeds *via* a slightly expanded transition-state ($m_{TSBI}$ = 1.2 ± 0.2 kJ mol^−1^ M^−1^). However, TS_BI_ is likely quite compact, as well. For example, a folding intermediate of the FF domain involving a relatively small structural change (66) where only five helix residues become disordered (relative to the folded state) has an m-value of ∼3 kJ mol^−1^ M^−1^ (6). Finally, $m_{TSEI}$, though poorly defined, is also small (−0.2 ± 0.9 kJ mol^−1^ M^−1^). Thus, the interconversion between the three compact states, E, B and I, proceeds *via* compact transition states.

## Conclusion

CPMG relaxation dispersion experiments have traditionally been used to study μs-ms timescale conformational exchange processes in proteins, enabling quantitative measures of the kinetics and thermodynamics of interconversion, and structural information about the molecular players involved (15, 58, 67). Here, we demonstrate the utility of CEST-based relaxation experiments, which, at least for the case of the L99A cavity mutant of T4 lysozyme, provide deeper insights into exchange processes than the CPMG approach. Unlike CPMG profiles of L99A T4L that are well-fit to a two-state model of chemical exchange, CEST data unequivocally establish a third exchanging conformer, providing the first experimental evidence in support of MD simulations (5, 45, 46, 47) that the FES of this protein is rugged in a region around the cavity. Further insight is likely to be obtained by performing additional relaxation experiments on suitable mutants, as has been done in studies elucidating the folding mechanisms of proteins (6, 8, 64, 68, 69). The L99A T4L system provides an example of how combined NMR and computational approaches can be used to obtain an in-depth understanding of protein dynamics, with CEST experiments playing a prominent role in quantifying rates and populations of a limited number of states and serving to validate results from simulations, which can then be used with confidence to map out further atomistic details of the process under investigation.

## Experimental procedures

### NMR samples

L99A T4L was expressed in *E coli* BL21(DE3) cells grown in M9 media supplemented with the appropriate precursors (70) and purified as described previously (51). The protein sample used for most of the studies contained ∼1.2 mM [U-^15^N; ^2^H; Ileδ1-^13^CH_3_; Leu, Val-^13^CH_3_/^12^CD_3_] (ILV) L99A T4L dissolved in 50 mM sodium phosphate, 25 mM NaCl, 2 mM NaN_3_, 2 mM EDTA, pH 5.5, (10% D_2_O) buffer. Samples used to validate the exchange model or to obtain urea m-values comprised ∼1.5 mM of [U-^15^N] L99A T4L dissolved in the above buffer but with either 8% V/V 2,2,2-trifluoroethanol (TFE) or the appropriate amount of urea (m-values).

### NMR experiments

All amide ^15^N CEST (20) and methyl ^13^C CEST (71, 72) experiments were carried out on a Bruker 700 MHz Avance III HD spectrometer equipped with a triple resonance cryogenically cooled probe. Details are provided in the Supporting information (Tables S1–S3). The strengths of the ^15^N or ^13^C $B_{1}$ fields applied in the *T*_*EX*_ periods during which exchange is quantified were calibrated using the nutation method (73).

### Generation of synthetic CPMG and CEST data

Synthetic ^15^N and ^13^C CPMG profiles were generated at static magnetic field ($B_{0}$) strengths of 11.7 T and 18.8 T by propagating the spin half Bloch-McConnell equations (74) for a sequence of π pulses in the context of the constant-time CW-CPMG experiment (16, 75, 76). Similarly, synthetic CEST data were generated at $B_{0}$ strengths of 18.8 T by propagating the Bloch-McConnell (74) equations for a *T*_*EX*_ period in the presence of weak *B*_*1*_ irradiation. Details can be found in the Supporting Information.

### Data analysis

NMR data were processed using the *NMRPipe* program (77), spectra were visualized and labeled using *Sparky* (78, 79), and, subsequently, peak intensities were extracted from pseudo-3D datasets using the program *PINT* (80). The software package *ChemEx* (https://github.com/gbouvignies/chemex) that numerically integrates the Bloch-McConnell equations (74) was used to fit various exchange models to the CEST and CPMG data and to extract the best-fit exchange parameters. Two (6, 20) and three-state (59) analyses of the CEST datasets were carried out as described previously, with details in the Supporting Information. Uncertainties in the fitted parameters were estimated using either bootstrap or Monte-Carlo approaches (81, 82), as described previously (59).

### Data availability

The CEST datasets analyzed during the current study are available from the corresponding authors upon reasonable request.

## Supporting information

The supporting information available free of charge from the journal website consists of a pdf file that contains additional experimental details, Supplementary figures and parameters (exchange rates, populations and chemical shifts) from the analysis of the CEST data (84).

## Conflict of interest

The authors declare that they do not have any conflicts of interest with the content of this article.
