# Supplementary material for: Using CEST NMR to discover previously unobserved states on the free energy surface of proteins: Application to the L99A cavity mutant of T4 lysozyme
Source: J Biol Chem. 2025 Nov 27;302(1):110989. doi: 10.1016/j.jbc.2025.110989 (PMC12805379; doi:10.1016/j.jbc.2025.110989)
Supplement: Supporting information [file mmc1.pdf]

Supporting Information

Using CEST NMR to Discover Previously Unobserved States on the Free Energy Surface of Proteins: Application to the L99A Cavity Mutant of T4 Lysozyme

Ved Prakash Tiwari[1], Nihar Pradeep Khandave[1], D. Flemming Hansen[2,3], Guillaume Bouvignies[4], Lewis E Kay[5,6,7,8*], Pramodh Vallurupalli[1,*]

[1]Tata Institute of Fundamental Research, Hyderabad.

36/P, Gopanpally Village, Serilingampally Mandal,

Ranga Reddy District, Hyderabad, Telangana 500107, India

[2]Department of Structural and Molecular Biology, Division of Biosciences, University College London, London WC1E 6BT, United Kingdom

[3]The Francis Crick Institute, London NW1 1AT, United Kingdom

[4]Chimie Physique Chimie du Vivant (CPCV), Departement de chimie, Ecole normale superieure, PSL University, Sorbonne Universite, CNRS, 75005 Paris, France

[5]Department of Molecular Genetics, University of Toronto, ON M5S 1A8, Canada

[6]Department of Biochemistry, University of Toronto, ON, M5S 1A8, Canada

[7]Department of Chemistry, University of Toronto, ON, M5S 3H6, Canada

[8]Program in Molecular Medicine, Hospital for Sick Children Research Institute, Toronto, ON, M5G 0A4, Canada

Experimental Procedures

Generation of synthetic CPMG and CEST data. To generate synthetic CPMG relaxation dispersion data of the type derived from the constant-time CW-CPMG experiment (1-3), the constant time exchange period T_{EX} was set to 20 ms and ν_{CPMG} (=1/(4τ_{CPMG})) varied from 50 Hz to 1000 Hz, where $2\tau_{CPMG}$ is the time between successive refocusing pulses in the CPMG pulse train. The effective transverse relaxation rate is given by $R_{2,eff}(\nu_{CPMG}) = \left(\frac{-1}{T_{EX}}\right) ln\left(\frac{I(\nu_{CPMG})}{I_0}\right)$ where $I(\nu_{CPMG})$ is the calculated intensity at the end of the T_{EX} period and I_0 is the intensity in the absence of the T_{EX} period (4). Gaussian random noise corresponding to the higher of 0.3 s^{-1} or 1.5 % of each $R_{2,eff}$ value was added to the calculated intensities. Synthetic ^{15}N CEST datasets (5) were generated for two different B_1 values: 17 Hz (T_{EX} = 450 ms) and 35 Hz (T_{EX} = 400 ms), with Gaussian noise corresponding to an uncertainty of 0.3 % in the intensities, *I*, added to the calculated *I* values. To construct synthetic ^{15}N CPMG and CEST datasets, 'reasonable' $\Delta\varpi_{AB}$ and $\Delta\varpi_{AC}$ values were obtained from the 58 $\Delta\varpi_{FI1}$ ($\Delta\varpi_{AB}$) and $\Delta\varpi_{FI2}$ ($\Delta\varpi_{AC}$) values generated from fits of CEST data from a previous study focussed on the folding trajectory of the A39G FF domain (6), setting $R_{2,A}$ = $R_{2,B}$ = $R_{2,C}$ = 10 s^{-1} and $R_{1,A}$ = $R_{1,B}$ = $R_{1,C}$ = 1 s^{-1}.

Analysis of CEST data. For the two-state analysis of the CEST data recorded on the [U-^{15}N; ^{2}H; Ileδ1-$^{13}CH_3$; Leu, Val-$^{13}CH_3$/$^{12}CD_3$] (ILV) L99A T4L sample (11.5 °C), ^{15}N CEST data from the amide sites of 18 residues (L7, R8, E11, I29, V75, I100, N101, F104, Q105, M106, E108, G110, A112, G113, A134, K135, S136, and T142) with $|\Delta\varpi_{EB}|$ > 2.0 ppm, and methyl ^{13}C CEST data from methyl sites of 5 residues (V103Cγ2, V111Cγ2, L121Cδ1, L118Cδ2 and L133Cδ2) with $|\Delta\varpi_{EB}|$ > 1.0 ppm were fit together. The global fitting parameters were the exchange rate ($k_{ex,EB}$) and the minor (B) state population (p_B), while the site-specific fitting parameters were the E (ϖ_E) and B (ϖ_B) state chemical shifts, the longitudinal relaxation rates of spins in state E ($R_{1,E}$) and the transverse relaxation rates of spins in the E ($R_{2,E}$) and B ($R_{2,B}$) states. In all analyses of CEST data carried out here the longitudinal relaxation rate of a given spin was assumed to be the same in all states. Some of the fits were carried out subject to the constraint $R_{2,E}$ = $R_{2,B}$, as described in the main text.

The three-state analysis of the 11.5 °C CEST data recorded on the [U-^{15}N; ^{2}H; Ileδ1-$^{13}CH_3$; Leu, Val-$^{13}CH_3$/$^{12}CD_3$] (ILV) L99A T4L sample was carried out using a protocol we recently described (7). Briefly the (relative) signs of the chemical shift differences between the minor state resonances ($\Delta\varpi_{BI}$) were obtained by analysing the (low B_1) ^{15}N (L7, R8, E11, I29, N101, M106, G110, A112, S136, T142) and methyl ^{13}C (V111Cγ2, L118Cδ2, L121Cδ1) CEST profiles recorded at 6, 11.5, 15 and 20 °C (Figure S2) in a two-state manner. The relative signs of $\Delta\varpi_{BI}$ are then obtained from the temperature dependencies of the fitted two-state $\Delta\varpi$ values ($\Delta\varpi_{2st}$) (Figure S2), with the absolute sign of a given $\Delta\varpi_{BI}$ value assumed to be the same as the

sign of the slope of the corresponding $\Delta\varpi_{2st}$ vs temperature profile. As shown in Figure S2 only the relative signs matter as inverting the signs of all $\Delta\varpi_{BI}$ values simply interchanges states I and B. Only sites for which $\Delta\varpi_{2st}$ monotonically increased or decreased with temperature were included in the three-state analysis, for which the global fitting parameters were the exchange rates ($k_{ex,EB}$, $k_{ex,EI}$, $k_{ex,BI}$) and the minor state populations (p_B, p_I) along with site-specific parameters which included chemical shifts of the three states (ϖ_E, ϖ_B, ϖ_I), and the longitudinal ($R_{1,E}$) and transverse ($R_{2,E}$) relaxation rates of spins in the E state since the relaxation rates of a given spin were assumed to be the same in all three states. Values of ($k_{ex,EB}$ = 270 ± 9 s^{-1}, $k_{ex,EI}$= 313 ± 45 s^{-1}, $k_{ex,BI}$ = 1645 ± 62 s^{-1}, p_B = 1.46 ± 0.01 %, and p_I = 0.28 ± 0.01 %; χ^2_{red} = 1) were obtained, with the sign of $\Delta\varpi_{BI}$ constrained to that obtained from the two-state analysis of CEST data over the 6 to 20 °C temperature range (Figure S2). The ^{15}N CEST profiles of N140 and the methyl ^{13}C CEST profiles of I150 Cδ1 recorded using high B_1 values (51.8, 72.6, and 129.6 Hz for ^{15}N; 49.1 and 98.2 Hz for ^{13}C) contained broad dips consistent with the I state. When these profiles were included in the analysis excellent quality fits were obtained overall, with exchange parameters similar to those reported above (χ^2_{red} = 1; $k_{ex,EB}$ = 297 ± 6 s^{-1}, $k_{ex,EI}$= 170 ± 37 s^{-1}, $k_{ex,BI}$ = 1667 ± 44 s^{-1}, p_B = 1.5 ± 0.01 %, and p_I = 0.22 ± 0.01 %). Simpler linear three-state models were also fit to the data (see main text), obtained by setting the appropriate exchange rate to 0 s^{-1}.

In the analysis of ^{15}N CEST profiles from [U-^{15}N] samples that contained urea or TFE, $\Delta\varpi_{EI}$ and $\Delta\varpi_{EB}$ values were initialised to those obtained above (Table S4). The three-state analysis of the ^{15}N CEST data from the sample containing 8% TFE resulted in good quality fits (χ^2_{red} = 1.16) with $k_{ex,EB}$ = 648 ± 47 s^{-1}, $k_{ex,EI}$= 160 ± 52 s^{-1}, $k_{ex,BI}$ = 4110 ± 172 s^{-1}, p_B = 2.2 ± 0.1 %, and p_I = 1.8 ± 0.1 %. In the analysis of ^{15}N CEST profiles to obtain urea m-values, the CEST data at all the urea concentrations were simultaneously fitted assuming that $\Delta\varpi_{EB}$ and $\Delta\varpi_{EI}$ do not depend on urea concentration (8), while ϖ_E was allowed to vary with urea concentration. This procedure resulted in good quality fits (χ^2_{red} = 1), with the exchange parameters reported in Table S5.

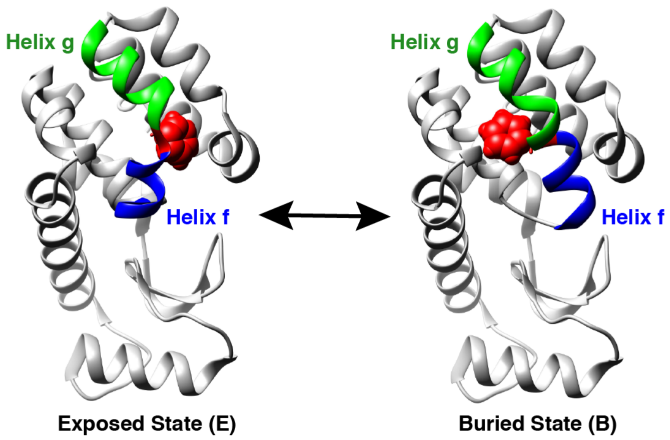

Figure S1 L99A T4L exchanges between a major state (Exposed state) in which Phe114 is exposed to solvent (PDB: 3DMV; (9)) and a minor state (Buried state) in which Phe114 is buried in the core of the protein (PDB: 2LCB; (10)). Phe114 (red) is shown using the CPK representation. In the buried state helix f (blue) and helix g (green) merge to form an extended helix.

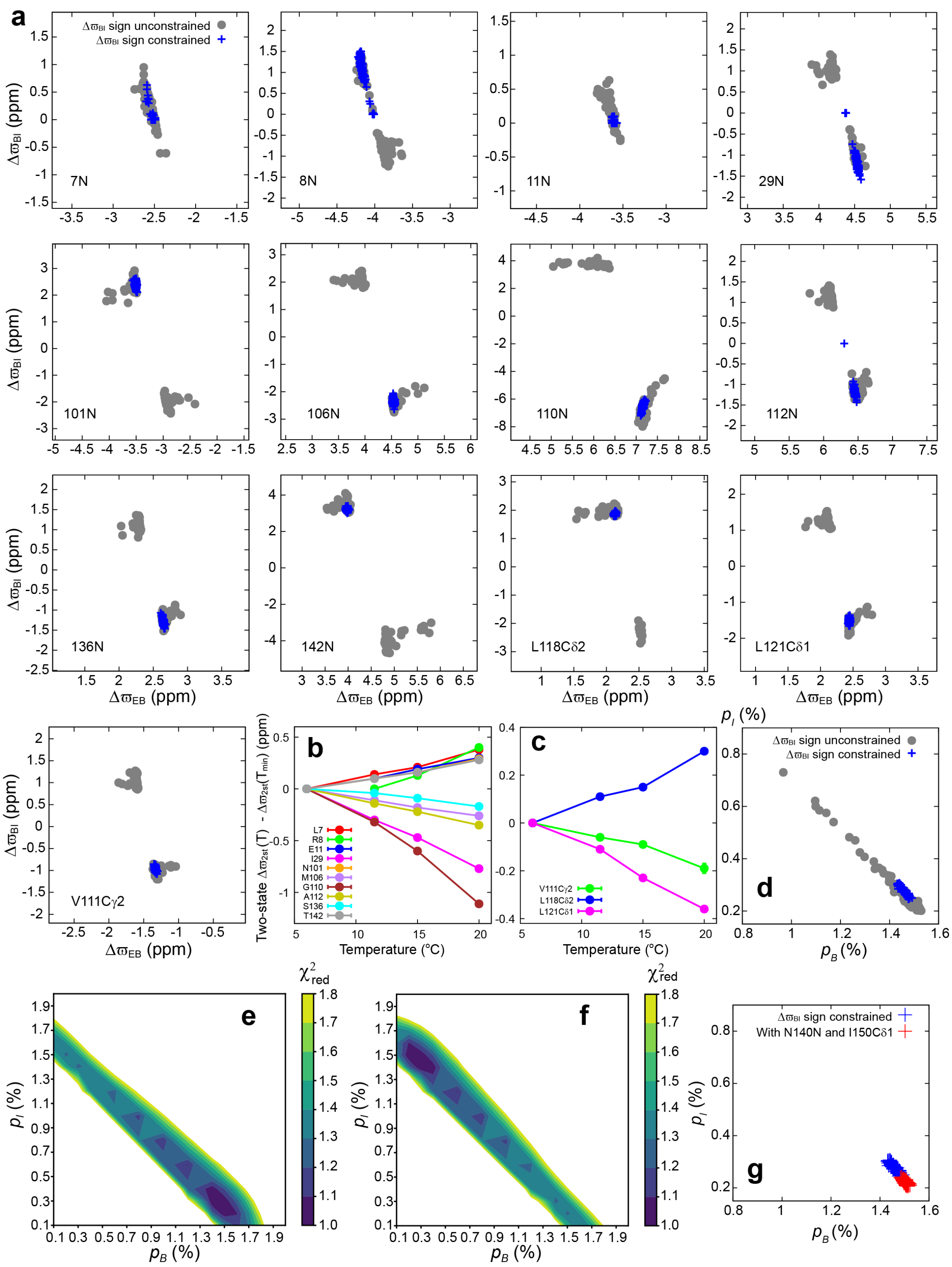

Figure S2 A unique L99A T4L three-state exchange model from amide ^{15}N and methyl ^{13}C CEST data. **(a)** $\Delta\varpi_{BI}$ and $\Delta\varpi_{EB}$ values for thirteen residues obtained from 100 bootstrap trials from the analysis of CEST data with (blue) and without (grey) constraining the sign of $\Delta\varpi_{BI}$. In the absence of constraints $\Delta\varpi_{BI}$ can adopt two values of approximately equal magnitude. Signs of $\Delta\varpi_{BI}$ are obtained from the temperature dependencies of two-state $\Delta\varpi$ values ($\Delta\varpi_{2st}$) (**b**,**c**). Assuming that $\Delta\varpi_{BI}$ values have the same signs as the slopes of the $\Delta\varpi_{2st}$ vs temperature plot (on a per-residue basis) leads to a consistent set of relative $\Delta\varpi_{BI}$ signs. **(d)** Including sign constraints on $\Delta\varpi_{BI}$ results in well defined p_B and p_I values (blue) while in the absence of

constraints p_B and p_I are not defined (grey). **(e,f)** χ^2 surface as function of p_B and p_I obtained by analysing the CEST data using a three-state triangular model. In **(e)** signs are as described above, while in **(f)** the signs of $\Delta\varpi_{BI}$ have been inverted i.e., sites for which the slope of the $\Delta\varpi_{2st}$ vs temperature plot is +ve have -ve $\Delta\varpi_{BI}$ values and vice versa. Equally good fits are obtained when the signs of $\Delta\varpi_{BI}$ are inverted, because the optimisation procedure interchanges the labels B and I (as expected), as seen from the fact that the plots in (**e**) and (**f**) are reflected about the diagonal. In **(e)** there is a global minimum with p_B >> p_I. **(g)** Including amide ^{15}N CEST profiles from N140 and $^{13}CH_3$ methyl CEST data from I150Cδ1 (where the I state dip is visible) in the three-state analysis also results in a minimum (red) consistent with that obtained previously (blue) from **(d)**.

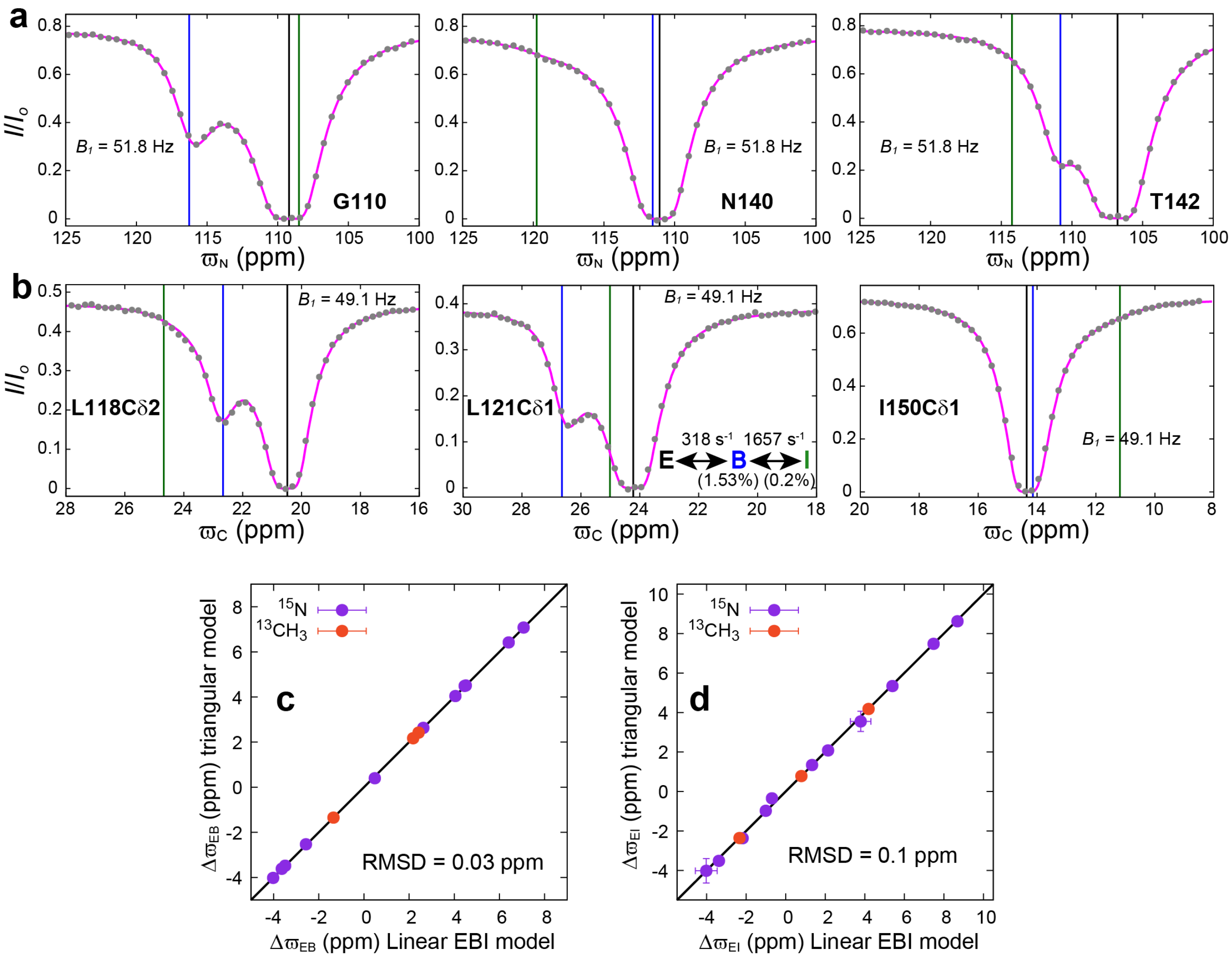

Figure S3 Linear (E↔B↔I) three-state analysis of the L99A T4L CEST data. Representative amide ^{15}N CEST profiles from G110, N140, and T142 **(a)** and methyl ^{13}C CEST profiles from L118Cδ2, L121Cδ1, and I150Cδ1 **(b)**. Experimental data points are shown using grey coloured circles while the magenta line was calculated using the best-fit parameters shown in the central panel in (**b**). The uncertainties in the I/I_0 values are smaller than the size of the filled circles. $\Delta\varpi_{EB}$ **(c)** and $\Delta\varpi_{EI}$ **(d)** values obtained from the E↔B↔I model are in excellent agreement with those obtained from the triangular model.

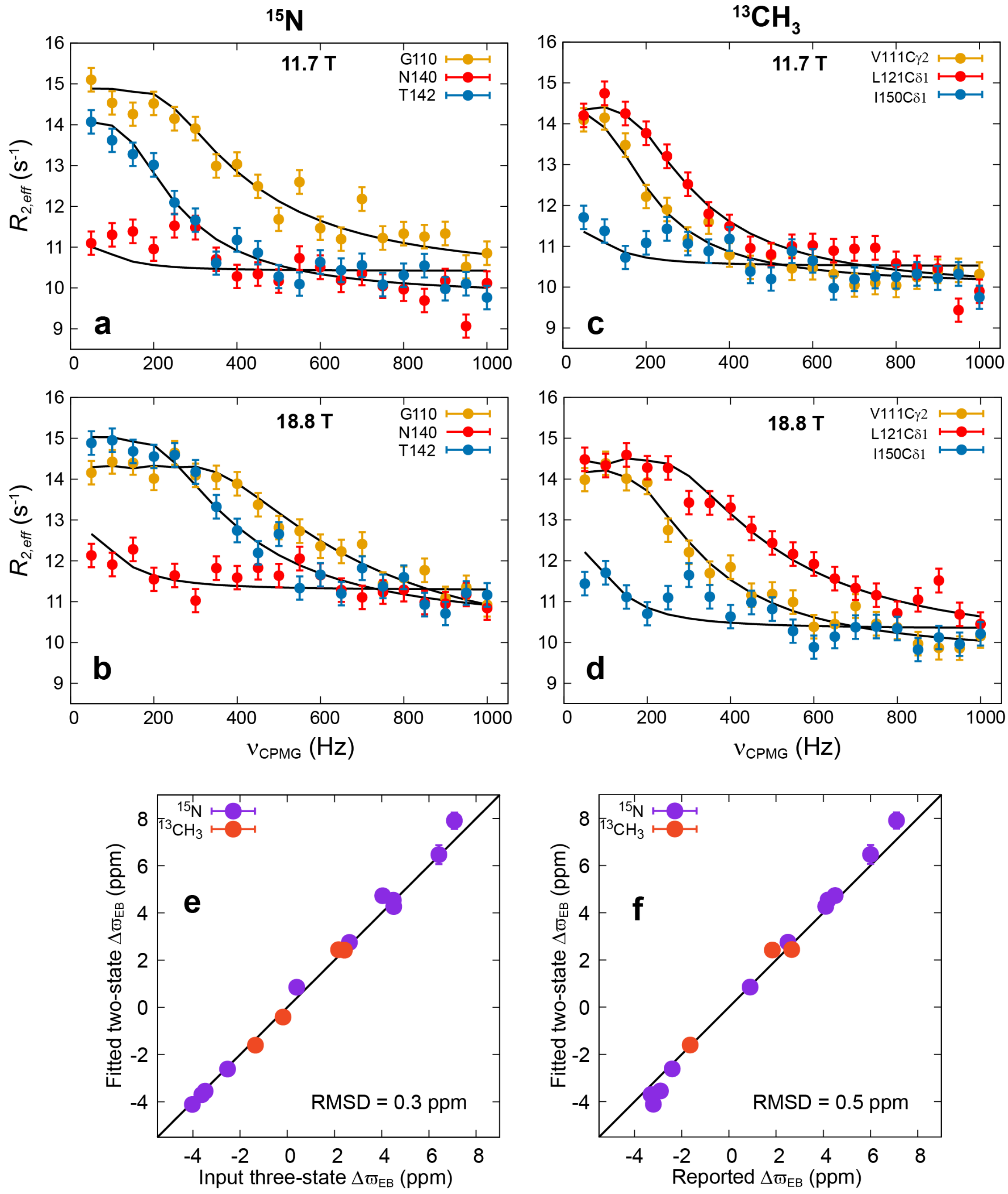

Figure S4 CPMG relaxation dispersion curves are insensitive to the presence of the I state. A two-state exchange model (black line) accounts (χ^2_{red} = 1.04) for synthetic three-state ^{15}N **(a, b)** and methyl ^{13}C CPMG **(c, d)** data. The synthetic data were generated using the best-fit three-state (triangular) exchange parameters ($k_{ex,EB}$ = 297 s^{-1}, $k_{ex,EI}$ = 170 s^{-1}, $k_{ex,BI}$ = 1667 s^{-1}, p_B = 1.5 %, and p_I = 0.22 %) and $\Delta\varpi_{EB}$ and $\Delta\varpi_{EI}$ values obtained from combined fits of ^{15}N and ^{13}C CEST data (Figure 3). Synthetic CPMG data points are represented using coloured circles while the two-state best-fits ($k_{ex,EB}$ = 318 ± 45 s^{-1} and p_B = 1.5 ± 0.3 %) are shown with black lines. **(e, f)** $\Delta\varpi_{EB}$ values obtained from two-state fits of the CPMG profiles agree well with the input $\Delta\varpi_{EB}$ values used to generate the CPMG profiles **(e)** as well as with those (Reported $\Delta\varpi_{EB}$) values previously obtained (10) from CPMG experiments **(f)**. As the shape of the CPMG relaxation dispersion curve is insensitive to the sign of the fitted $\Delta\varpi$ value (11,12), the sign of the fitted $\Delta\varpi$ values (Fitted two-state $\Delta\varpi_{EB}$) were assumed to be the same as those obtained from the CEST data analysis.

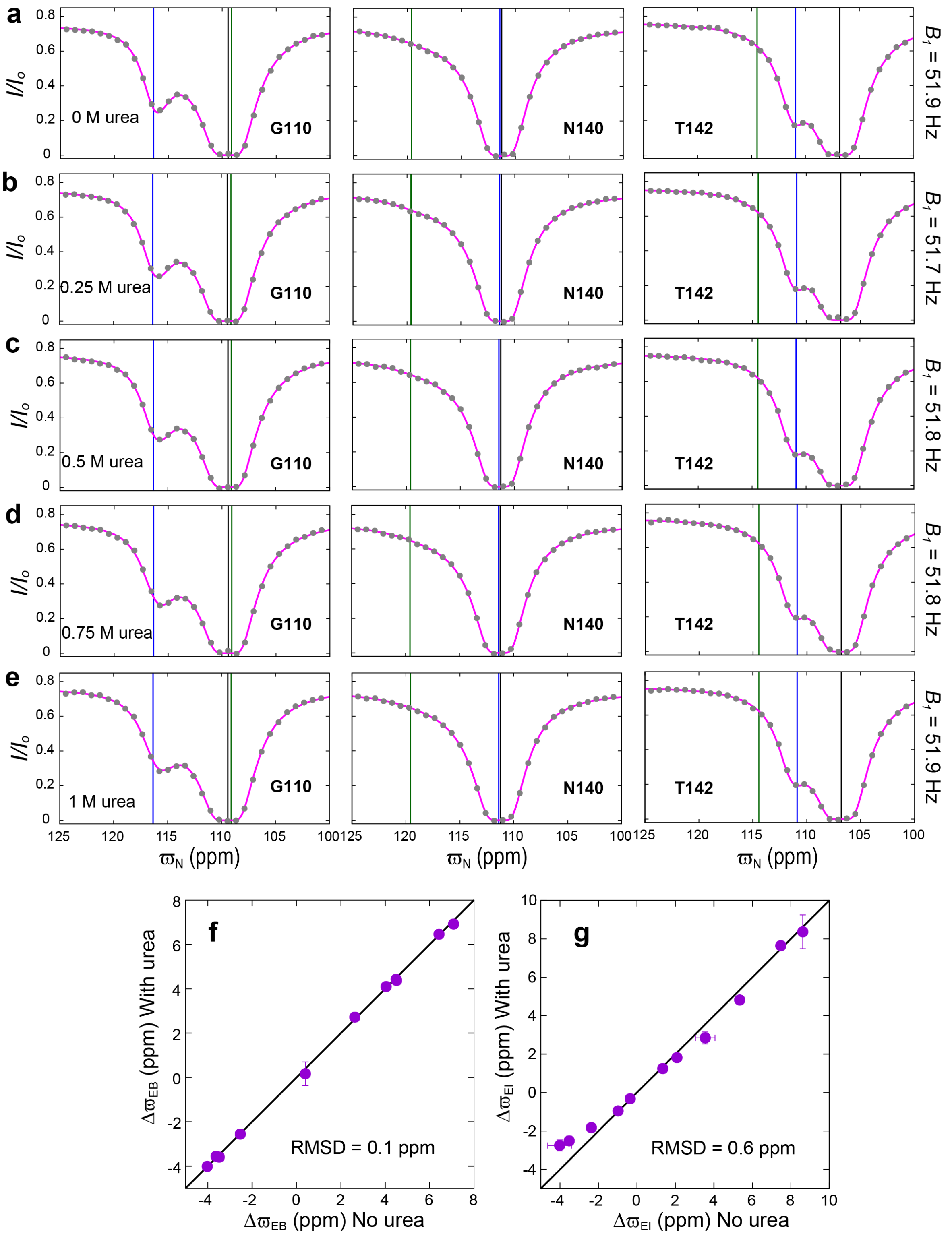

Figure S5 ^{15}N CEST data recorded at various urea concentrations to obtain urea m-values (11.5 ^{o}C). Representative ^{15}N CEST data and three-state fits (from residues G110, N140 and T142) recorded using [U-^{15}N] L99A T4L samples containing 0.0 **(a)**, 0.25 M **(b)**, 0.5 M **(c)**, 0.75 M **(d)** and 1.0 M **(e)** urea. The experimental data points are shown as grey circles while the magenta line was calculated using the (three-state) best-fit parameters (Table S5). The uncertainties in the I/I_0 values are smaller than the size of the filled circles. The vertical lines are drawn at the best-fit ϖ_E (black), ϖ_B(blue), and ϖ_I (green) values obtained from

the three-state fits. Data collected at all the urea concentrations were analysed simultaneously with $\Delta\varpi_{EB}$ and $\Delta\varpi_{EI}$ assumed to be urea concentration independent while ϖ_E was allowed to vary with urea concentration. Comparison of amide ^{15}N $\Delta\varpi_{EB}$ (**f**) and $\Delta\varpi_{EI}$ (**g**) values obtained in the analysis of the urea concentration series with those obtained by analysing ^{15}N and ^{13}C CEST data in the absence of urea.

Temperature (°C)	Experiment	B_1 (Hz)	T_{EX} (ms)	Range (Hz)	Step-size (Hz)	Carrier (ppm)
11.5	^{15}N CEST	18.1	450	±1260	20	119.707
		33.2	450	±1250	25	119.707
		51.8	400	±1400	40	119.707
		72.6	375	±1400	50	119.707
		129.6	350	±1760	55	119.707
	^{13}C CEST	16.2	475	-1500 to 2000	20	17.868
		32.4	450	-1620 to 2130	30	17.868
		49.1	425	-1650 to 2150	40	17.868
		98.2	375	-1800 to 2260	70	17.868
6.0	^{15}N CEST	31.2	450	±1260	30	119.765
		67.5	375	±1400	50	119.765
	^{13}C CEST	27.1	450	-1620 to 2130	30	17.927
15.0	^{15}N CEST	25.9	450	±1350	25	119.682
		51.8	400	±1400	40	119.682
	^{13}C CEST	48.9	400	-1650 to 2150	40	17.846
20.0	^{15}N CEST	36.5	330	±1350	30	119.627
		52.1	325	±1400	40	119.627
	^{13}C CEST	49.7	340	-1650 to 2150	40	17.789

Table S1 Details of amide ^{15}N and methyl ^{13}C CEST experiments(5,13) using the sample containing ~1.2 mM [U-^{15}N; ^{2}H; Ileδ1-$^{13}CH_3$; Leu, Val-$^{13}CH_3$/$^{12}CD_3$] (ILV) L99A T4L dissolved in 50 mM sodium phosphate, 25 mM NaCl, 2 mM NaN_3, 2 mM EDTA, pH 5.5, (10% D_2O) buffer. The B_1 irradiation was applied for a time T_{EX} over a range of offsets around the carrier, as indicated (Range), and Step-size is the spacing between adjacent offsets. A reference plane without the T_{EX} delay was also recorded. The 11.5 ^{o}C datasets were analysed using both two and three-state exchange models while the 6.0, 15.0 and 20.0 ^{o}C datasets along with the 11.5 ^{o}C dataset were analysed using a two-state model to obtain the signs of $\Delta\varpi_{BI}$ values.

Temperature (°C)	Experiment	B_1 (Hz)	T_{EX} (ms)	Range (Hz)	Step-size (Hz)	Carrier
15	^{15}N CEST	17.5	450	±1190	20	119.689
15	^{15}N CEST	51.4	350	±1400	40	119.689
15	^{15}N CEST	102.9	300	±1760	55	119.689

Table S2 Details of the amide ^{15}N CEST experiments (5) carried out to establish the robustness of the CEST derived ϖ_I values. The sample contained ~1.5 mM [U-^{15}N] L99A T4L dissolved in 50 mM sodium phosphate, 25 mM NaCl, 2 mM NaN_3, 2 mM EDTA, 8% (v/v) TFE, pH 5.5, (5% D_2O) buffer. During the exchange time, T_{EX}, B_1 irradiation was applied over a range of offsets around the carrier, with a spacing given by Step-size. A reference plane without the T_{EX} delay was also recorded.

Temperature (°C)	Experiment	Urea Concentration (M)	B_1 (Hz)	T_{EX} (ms)	Range (Hz)	Step-size (Hz)	Carrier
11.5	^{15}N CEST	0.0	26.0	480	±1155	35	119.706
			51.9	450	±1400	56	119.706
			114.2	350	±1680	70	119.706
11.5	^{15}N CEST	0.25	25.9	480	±1155	35	119.706
			51.7	450	±1400	56	119.706
			113.7	350	±1680	70	119.706
11.5	^{15}N CEST	0.5	25.9	480	±1155	35	119.706
			51.8	450	±1400	56	119.706
			114.0	350	±1680	70	119.706
11.5	^{15}N CEST	0.75	26.0	480	±1155	35	119.706
			51.9	450	±1400	56	119.706
			114.2	350	±1680	70	119.706
11.5	^{15}N CEST	1.0	25.9	480	±1155	35	119.706
			51.8	450	±1400	56	119.706
			114.0	350	±1680	70	119.706

Table S3 Details of amide ^{15}N CEST experiments (5) carried out to derive urea m-values for E, B, and I, and the associated transition states using the three-state triangular exchange model. Samples were comprised of ~1.5 mM [U-^{15}N] L99A T4L dissolved in 50 mM sodium phosphate, 25 mM NaCl, 2 mM NaN_3, 2 mM EDTA, pH 5.5, (5% D_2O) buffer with an amount of urea as indicated. B_1 irradiation was applied during the exchange time, T_{EX}, over a range of offsets around the carrier, spaced at intervals of Step-size. A reference plane without the T_{EX} delay was also recorded.

Residue	Site	ϖ_E (ppm)	$\Delta\varpi_{EB}$ (ppm)		$\Delta\varpi_{EI}$ (ppm)	
			Value	Uncertainty	Value	Uncertainty
V111	Methyl $^{13}C\gamma 2$	24.28	-1.35	0.20	-2.36	0.20
L118	Methyl $^{13}C\delta 2$	20.48	2.17	0.20	4.18	0.20
L121	Methyl $^{13}C\delta 1$	24.22	2.42	0.20	0.78	0.20
I150	Methyl $^{13}C\delta 1$	14.35	-0.18	0.20	-3.14	0.23
L7	Amide ^{15}N	118.93	-2.53	0.20	-2.37	0.20
R8	Amide ^{15}N	124.22	-4.02	0.20	-4.02	0.62
E11	Amide ^{15}N	118.78	-3.62	0.20	-3.52	0.20
I29	Amide ^{15}N	129.78	4.49	0.20	3.55	0.51
N101	Amide ^{15}N	119.36	-3.48	0.20	-0.98	0.20
M106	Amide ^{15}N	113.12	4.51	0.20	2.08	0.20
G110	Amide ^{15}N	109.21	7.08	0.20	-0.35	0.22
A112	Amide ^{15}N	117.90	6.42	0.20	5.34	0.20
S136	Amide ^{15}N	113.08	2.63	0.20	1.34	0.20
N140	Amide ^{15}N	111.06	0.40	0.20	8.62	0.20
T142	Amide ^{15}N	106.77	4.04	0.20	7.48	0.20

Table S4 ϖ_E, $\Delta\varpi_{EB}$ and $\Delta\varpi_{EI}$ values obtained by analysing the 11.5 ^{o}C CEST data from the ~1.2 mM [U-^{15}N; ^{2}H; Ileδ1-$^{13}CH_3$; Leu, Val-$^{13}CH_3$/$^{12}CD_3$] (ILV) L99A T4L sample (50 mM sodium phosphate, 25 mM NaCl, 2 mM NaN_3, 2 mM EDTA, pH 5.5, 10% D_2O, buffer). A triangular three-state model was used. Uncertainties were estimated using a bootstrap procedure (14,15) with 100 trials. The minimum value of the reported uncertainty in $\Delta\varpi_{EB}$ and $\Delta\varpi_{EI}$ was set to 0.2 ppm.

Urea Concentration [M]	$k_{ex,EB}$ (s^{-1})	$k_{ex,EI}$ (s^{-1})	$k_{ex,BI}$ (s^{-1})	p_B (%)	p_I (%)
0.0	236.9 ± 9	355.1 ± 65	1464.9 ± 55	1.57 ± 0.02	0.24 ± 0.01
0.25	265.9 ± 8	297.0 ± 55	1607.5 ± 49	1.56 ± 0.01	0.26 ± 0.01
0.5	283.8 ± 9	285.3 ± 54	1799.1 ± 58	1.49 ± 0.01	0.26 ± 0.01
0.75	303.4 ± 12	257.8 ± 65	2006.4 ± 66	1.43 ± 0.02	0.28 ± 0.02
1.0	322.4 ± 18	303.2 ± 88	2204.3 ± 64	1.36 ± 0.02	0.28 ± 0.02

Table S5 Exchange parameters obtained from a triangular three-state analysis of amide ^{15}N CEST data recorded at 11.5 ^{o}C (Table S3) using ~1.5 mM [U-^{15}N] L99A T4L samples dissolved in 50 mM sodium phosphate, 25 mM NaCl, 2 mM NaN_3, 2 mM EDTA, pH 5.5, (5% D_2O) buffer, with the amount of urea indicated. ^{15}N CEST data at all the urea concentrations were simultaneously analysed assuming that $\Delta\varpi_{EB}$ and $\Delta\varpi_{EI}$ are invariant to the addition of urea, while ϖ_E was allowed to vary with urea concentration. Uncertainties were estimated using a bootstrap procedure with 50 trials.

References

1. Hansen, D. F., Vallurupalli, P., and Kay, L. E. (2008) An improved 15N relaxation dispersion experiment for the measurement of millisecond time-scale dynamics in proteins. *J Phys Chem B* **112**, 5898-5904

2. Skrynnikov, N. R., Mulder, F. A., Hon, B., Dahlquist, F. W., and Kay, L. E. (2001) Probing slow time scale dynamics at methyl-containing side chains in proteins by relaxation dispersion NMR measurements: application to methionine residues in a cavity mutant of T4 lysozyme. *J Am Chem Soc* **123**, 4556-4566

3. Vallurupalli, P., Scott, L., Williamson, J. R., and Kay, L. E. (2007) Strong coupling effects during X-pulse CPMG experiments recorded on heteronuclear ABX spin systems: artifacts and a simple solution. *J Biomol NMR* **38**, 41-46

4. Mulder, F. A., Skrynnikov, N. R., Hon, B., Dahlquist, F. W., and Kay, L. E. (2001) Measurement of slow (micros-ms) time scale dynamics in protein side chains by (15)N relaxation dispersion NMR spectroscopy: application to Asn and Gln residues in a cavity mutant of T4 lysozyme. *J Am Chem Soc* **123**, 967-975

5. Vallurupalli, P., Bouvignies, G., and Kay, L. E. (2012) Studying "invisible" excited protein States in slow exchange with a major state conformation. *J Am Chem Soc* **134**, 8148-8161

6. Tiwari, V. P., Toyama, Y., De, D., Kay, L. E., and Vallurupalli, P. (2021) The A39G FF domain folds on a volcano-shaped free energy surface via separate pathways. *Proc Natl Acad Sci U S A* **118**

7. Tiwari, V. P., De, D., Thapliyal, N., Kay, L. E., and Vallurupalli, P. (2024) Beyond slow two-state protein conformational exchange using CEST: applications to three-state protein interconversion on the millisecond timescale. *J Biomol NMR*

8. De, D., Thapliyal, N., Prakash Tiwari, V., Toyama, Y., Flemming Hansen, D., Kay, L. E., and Vallurupalli, P. (2024) Mapping the FF domain folding pathway via structures of transiently populated folding intermediates. *Proc Natl Acad Sci U S A* **121**, e2416682121

9. Liu, L. J., Baase, W. A., and Matthews, B. W. (2009) Halogenated Benzenes Bound within a Non-polar Cavity in T4 Lysozyme Provide Examples of I---S and I--Se Halogen-bonding. *Journal of Molecular Biology* **385**, 595-605

10. Bouvignies, G., Vallurupalli, P., Hansen, D. F., Correia, B. E., Lange, O., Bah, A., Vernon, R. M., Dahlquist, F. W., Baker, D., and Kay, L. E. (2011) Solution structure of a minor and transiently formed state of a T4 lysozyme mutant. *Nature* **477**, 111-114

11. Skrynnikov, N. R., Dahlquist, F. W., and Kay, L. E. (2002) Reconstructing NMR spectra of "invisible" excited protein states using HSQC and HMQC experiments. *J Am Chem Soc* **124**, 12352-12360

12. Gopalan, A. B., and Vallurupalli, P. (2018) Measuring the signs of the methyl ^{1}H chemical shift diferences between major and ‘invisible’ minor protein conformational states using methyl ^{1}H multi‐quantum spectroscopy. *J Biomol NMR* **70**, 187-202

13. Bouvignies, G., and Kay, L. E. (2012) A 2D C-13-CEST experiment for studying slowly exchanging protein systems using methyl probes: an application to protein folding. *J Biomol NMR* **53**, 303-310

14. Press, W. H., Flannery, B. P., Teukolsky, S. A., and Vetterling, W. T. (1992) *Numerical Recipes in C. The Art of Scientific Computing* Second Edition ed., Cambridge University Press, Cambridge (UK)

15. Choy, W. Y., Zhou, Z., Bai, Y., and Kay, L. E. (2005) An 15N NMR spin relaxation dispersion study of the folding of a pair of engineered mutants of apocytochrome b562. *J Am Chem Soc* **127**, 5066-5072
